# Examining Implementation of Tobacco Control Policy at the District Level: A Case Study Analysis from a High Burden State in India

**DOI:** 10.1155/2016/4018023

**Published:** 2016-01-03

**Authors:** Divya Persai, Rajmohan Panda, Adyya Gupta

**Affiliations:** ^1^Public Health Foundation of India, New Delhi 110019, India; ^2^School of Public Health and Australian Research Centre for Population Oral Health, University of Adelaide, Adelaide, SA 5005, Australia

## Abstract

*Introduction.* While extensive scientific evidence exists on the tobacco epidemic, a lack of understanding of both policies and their appropriate way of implementation continues to hinder effective tobacco control. This is especially so in the developing countries such as India. The present study aims to understand current implementation practices and the challenges faced in mainstreaming tobacco control policy and program.* Methods.* We chose a qualitative study design to conduct the case analysis. A total of 42 in-depth interviews were undertaken with seven district officials in six districts of Andhra Pradesh. A conceptual framework was developed by applying grounded theory for analysis. Analysis was undertaken using case analysis approach.* Results and Discussion.* Our study revealed that most program managers were unfamiliar with the comprehensive tobacco control policy. Respondents have an ambiguous opinion regarding integration of tobacco control program into existing health and development programs. Respondents perceive lack of resources, low prioritization of tobacco control, and lack of monitoring and evaluation of smoke-free laws as limiting factors affecting implementation of tobacco control policy.* Conclusion.* The findings of this study highlighted the need for a systematic, organized action plan for effective implementation of tobacco control policy and program.

## 1. Introduction

Tobacco use is the world's leading preventable cause of death. It has been the cause of 5.4 million deaths annually and the toll is expected to account for 10% of all deaths globally by 2015 [1]. In the 21st century, tobacco's toll is expected to be responsible for 1 billion deaths globally [2]. It has been estimated that tobacco smoking was a single most preventable cause of 1 million deaths a year in India in early 2010 [3]. To curb this growing menace, National Tobacco Control Program (NTCP) was piloted in India in 2007-2008 to strengthen the implementation of tobacco control policy. The program is currently implemented in 21 states and 42 districts in India. The NTCP in its current form has a national, state, and district tobacco control cell. The national cell is responsible for planning of activities, policy formulation, and monitoring and evaluation [4].

The district tobacco control cell is responsible for overall planning, implementation, and monitoring of different activities and achievement of physical and financial targets planned under the program in the district. The NTCP outlines the involvement of district level health functionaries in implementation of various tobacco control policy measures which includes enforcement and monitoring of implementation of tobacco control laws [4]. It also envisages that district tobacco control cell should develop awareness and mass media campaigns for awareness generation on the harmful effects of tobacco. However, the district level health system lacks sufficient capacity to mainstream tobacco control in health and developmental activities [5]. It has also been recognized that there is a lack of effective monitoring mechanism in about half of the states where NTCP is under implementation [6]. In order to manage the enforcement and involve the health workforce in tobacco control, engagement of key decision-makers at district level is essential.

While extensive scientific evidence exists on the tobacco epidemic, a lack of understanding of both policies and their implementation continues to hinder effective tobacco control. This is especially so in the context of developing countries such as India [7]. Studies, which explored stakeholders' responses across areas of tobacco control policies in Thailand and Zimbabwe, recommend a need to expand the number of policy studies in developing countries so as to truly understand the environments in which tobacco control implementation takes place [8]. Understanding health policy makers and program managers' opinions towards implementation of tobacco control policy is essential since they play a key role in decision-making [9]. Understanding their perspectives can help strengthen the implementation and suggest midway corrections in the policies aiming to strengthen the diagonal integration of tobacco control in health systems [10, 11]. We undertook a study amongst senior health program managers to understand current implementation practices and the challenges faced in mainstreaming tobacco control programs in the state of Andhra Pradesh. The study was conducted in six districts of a southern state, Andhra Pradesh (AP), in India. Geographically, AP is the fourth largest state in India. It is one of the largest tobacco producing states with a high burden of tobacco use in India [12]. According to the GATS (Global Adult Tobacco Survey, 2009-10) data, 48% of males and 20% of females consume tobacco in some or the other form in the state of AP [13].

## 2. Methods

A qualitative study design was employed to conduct the case study analysis. Case studies are in-depth investigations of a single instance of a phenomenon in its real-life context. Program managers' perceptions and beliefs about tobacco control as a part of a health program constituted the case. Qualitative design helped us to explore in-depth understanding of the program managers' perspective regarding the success and challenges faced by them in implementation of tobacco control policy and program.

### 2.1. Study Settings and Respondents

The study was conducted in 6 districts of Andhra Pradesh. A total of 42 in-depth interviews were undertaken where seven district officials were interviewed from each district. A purposive sampling technique was employed with a conscious selection of certain subjects who could provide in-depth information on the topic of interest. We adopted a snowball approach asking each respondent at the end of the interview to recommend further respondents. The respondents were drawn from the health departments at the district level and were comprised of the following:

- Senior program heads responsible for different health programs like district tuberculosis control and reproductive and child health program.
- Senior program officers in charge of the district units of the National Rural Health Mission (NRHM), a flagship program of the Government of India that provides managerial and program support to ongoing health programs in the state of Andhra Pradesh in India.

Divergent constituencies belonging to different health programs were chosen for this study so that differences in their approach towards the implementation of tobacco control in the districts could be captured meaningfully.

### 2.2. Study Tool

A discussion guide was used as the research tool for this study. The discussion guide consists of several themes, questions, and also probes for further inquiry wherever required (Box 1).

### 2.3. Data Collection

Field investigators who had prior experience in collecting qualitative data were trained in collecting data in local language. They worked in close proximity with the investigating team. Audio tapes were used to record the interviews, wherever permission was granted. Summary sheets were used to capture nonverbal information shared by the respondent during the course of the interview. After the interviews were completed, transcripts were generated by translating from local language into English taking care of subtle nuances and the meaning of specific words. While the fieldwork was going on, the initial few transcripts were closely reviewed in the field to assess the relevance and comprehensiveness of the information obtained and corrections were made. The first author accompanied the investigator in the first few interviews to guide the investigation.

Table 1 describes the key themes which emerged during case analysis. The subjectivity inherent in the case study was reduced through prudent selection of the case study interviewees, an interview process, and a systematically documented process for recording, transcribing, and interpreting the data. Internal validity was established by the use of cross case analysis and pattern matching techniques. Diagrams, illustrations, and data matrices were built to demonstrate the internal consistency of the information collected.

### 2.4. Data Analysis

The field notes of the pilot study were reviewed for accuracy and completion; changes were made to the guide and tested in a neutral district, following which the fieldwork was initiated in the intervention districts. The data obtained from the pilot transcript was used as the basis for generating new codes. The new codes were added to the previous codebook. The discussion guide used for this study listed down all the questions/themes that need to be addressed in interviews (which were transcribed by subject experts and used as transcripts). The data consist of taped recorded conversations and memos of field investigators. A conceptual framework was developed by applying Grounded theory for analysis, making an attempt to not only contribute to the existing knowledge but also highlight new emerging theories inductively. Grounded theory was considered appropriate in the present study as it applies a systematic procedure to generate theory or insights describing a phenomenon which is grounded in the views expressed by study participants [14]. Analysis in the present study is similar to the analysis done in Grounded theory studies which is typically guided by the constant comparative method, in which verbatim quotations are catalogued into their essential concepts by use of codes developed iteratively to reflect the data.

We adopted a “case analysis” approach in this study where we analyzed the data in 2 stages as follows.

**Stage 1 .**

The process was initialized with organizing the interviews into groups based on their similarities in responses for each specific interview question as noted in Box 1. These groups were called “Cases.” A framework was developed using a thematic code based on which the data were coded. Retrievals and comparisons of the coded data enabled pulling out the emerging themes and subthemes inductively. Each transcript was reviewed thoroughly for their similarities and dissimilarities in their perspectives and experiences across each theme. This design allowed us to explore, analyze, and understand perceptions of senior health program managers regarding tobacco control implementation and its possible integration within the capacity of other programs.

**Stage 2 .**

Patterns of similarities and dissimilarities were searched across the cases. The transcripts were reviewed as multiple comparative cases; the coding was guided by the perspectives and experiences of one group of officials compared to the others as per a specific theme. The codes that generated from the data during analysis were revisited and revised and the codebook was updated accordingly. Similar codes clubbed together under families and themes were generated. Additional review of the coded data and reflection on the initial conceptualization led to refinement of the concepts used to describe the program manager's perspectives on the implementation of tobacco control in the districts. This provided more meaning to the data and helped in its interpretation.

In describing the data, the terms “few,” “majority,” and “most” are used to represent approximately less than 40%, 40–80%, and more than 80%, respectively, of proportions of respondents in the study. ATLAS.ti 7 software was used for analysis.

### 2.5. Ethical Considerations

Informed consent was taken from the respondents. All respondents were asked if they are comfortable with the audio recording of their interviews, and care was taken not to intrude upon the schedule of the respondents by seeking prior appointments for the interview. A written permission was also obtained from the state department and each of the district administrations for the study. The study was conducted from March to April 2011. The study was approved by the Institutional Ethics Committee (IEC number 65/60).

## 3. Findings and Discussion

Case study analysis in health system research helps in understanding the current levels of knowledge and awareness of key stakeholders involved in decision-making processes. In the present case study analysis, intensive and rigorous two staged case by case analysis was conducted to explore in depth understanding and comparison of program manager's perceptions on each theme. The first level of “case analysis” methodology design enabled us to group similar and dissimilar responses to the interview questions and form cases as described in Box 1. This gave us an overview of the responses of the program managers about each specific question and helped us in exploring the emerging pattern. Further, the group analysis in the case analysis approach allowed us to segregate cases into separate groups based on their similarities and dissimilarities, thereby analyzing and understanding perceptions of the program managers on various aspects. Finally, four broad themes emerged which reflected the different perceptions of the program managers on implementation of tobacco control policy.

The findings are broadly categorized into four different themes and are discussed as follows.

*(1) Implementation of Tobacco Control Policy.* Program managers highlighted several potential solution levers to strengthen the enforcement of existing tobacco control policy. Awareness generation among policy makers was the most common and cross cutting suggestion reported by the majority of respondents. A majority of them emphasized enhancing enforcement of tobacco control policy by including various information, education, and communication channels like television, media, advertisements, health warning, and health education. Respondents opined that multifaceted educational strategies in conjunction with community-based campaigns will bring substantial change in the trajectory of tobacco use.

*Public awareness should be improved. IEC, information, education and communication should be properly implemented… Then this program (NTCP) will be successful.*

A majority of program managers mentioned that involvement of policy makers will prioritize the implementation of tobacco control policy. Respondents mentioned that policy makers should be sensitized to ensure monitoring of tobacco control policy.

*Everyone (policy makers and community) should help and play their role in controlling tobacco epidemic… it is not only the people who are working in health programs or in tobacco control….*

Lastly, it was suggested by few program managers that changes in the fiscal policy and cost of tobacco products may also bring a visible difference in its use.

*Cost of tobacco products should be increased so that they (people) can stop (consuming it)….*

Tobacco control campaigns, which primarily utilized mass media to educate and inform the public, have been effective in promoting antismoking, thereby reducing tobacco prevalence in high-income countries [15, 16]. Similar evidence from low- and middle-income countries is limited but emerging [17]. Consistent with the findings of study conducted by Tompson et al. in Lao PDR in 2009 [18], program managers in our study emphasized positive role of mass media and information, education, and communication in effective implementation of tobacco control policy.

*(2) Effectiveness of Smoke-Free Policies.* Most cases across all districts of Andhra Pradesh were familiar with the law on smoke-free public places and its regulations (fines and penalties).

*If they (people) smoke, they (people) have to pay a fine.*

However, there were a few cases that were unaware of the policy per se but were familiar with the norms of the policy.

*Yes, it (policy) is there, but I don't have that much idea about that policy, (it) should not (be) sold near school(s), no smoking in public places like bus stop, etc.*

Few cases viewed the lack of public support from the masses as an important barrier to implementation of the law. They emphasized the combined effort of government and the people which would result in the country getting rid of tobacco menace. They acknowledged that health care providers play a crucial role in tobacco control as they are in direct contact with patients. The findings of our study are consistent with other studies conducted in India and across the world which underscore the lack of public support as a barrier to implementation of tobacco control policy [9, 19].

*Can't say much about it [Implementation] because the policy [No smoking policy] are just policies because people just read it and forget…. They [People] should themselves follow it first [Policy].*

The smoke-free policy in public places came into enforcement in India in 2008 as per Section 4 of COTPA (Cigarettes and Other Tobacco Products-Prohibition of Advertisement and regulation of trade, commerce, production, supply, and distribution Act, 2003) [4]. This policy included various enforcement mechanisms like monetary fines and penalties. Our findings reveal that most of the cases (about 70%) were not familiar with the comprehensive policy: only few (about 30%) were aware of the policy of prohibition of smoking in public places, on public transports, and in places like movie theatres.

*(3) Integration of Tobacco Control Program into Existing Health Programs.* Most of the cases view integrating tobacco control with other health and development programs to be a valuable tool. Respondents mentioned that this will also help in simplifying technical and financial support. For instance,

*The issue [Tobacco issue] has to integrate with NRHM, RNTCP, We [Stakeholder] have to tell them to do this [Tobacco control] program with that [NGO's own program] program, we have to give some instructions to them (NGO's).*

Few other cases were concerned that integrating tobacco control with other health programs would lead to dilution of the message as it would then become a low priority within the larger programs. Respondents stated the following:

*If we give (do) 10 programs likes that (together) it (tobacco control) gets diluted.*

*It (tobacco control) should be a separate program not integrated.*

*They (NRHM) are unable to do properly, so how can we (stakeholders) integrate with it?*

Thus, mixed reactions were observed on the effectiveness of integration of tobacco control program with other health programs. This is expected as the health programs that these program managers implement and overlook are different in design, size, and scope of implementation at the district level. The actual capacity and implementation of these programs also vary in different districts depending upon resources. Senior managers from NRHM (National Rural Health Mission) and RNTCP (Revised National Tuberculosis Control Program) continued to address the need for integration of tobacco cessation under large program. Many studies have revealed the importance of integrating tobacco control into health and development agendas [20, 21]. A situational analysis conducted in Andhra Pradesh in 2010 also recommended integration to be highly effective as large programs are well established and have robust functioning mechanisms already in place [5]. World Health Organization (2008) also recommended that tobacco control intervention should be incorporated and integrated into primary health care services and programs [21].

Moving tobacco control toward systems approach management envisages integrating the advantages of traditional and systems' views so as to enable health care providers to deal flexibly with myriad organizational contexts [22]. One way of doing so is by linking them to the existing health services and enhancing the outreach [23]. Interconnected actions are required across different levels for prevention and surveillance while continuing to work for communicable diseases, nutrition, and maternal and child health issues.

*(4) Organizational Barriers for Implementation of Tobacco Control Policy and Program*

*(4.1) Tobacco Control: Lack of Prioritization.* It is evident from most of the transcripts that tobacco control was not on a high priority agenda of the health department as compared to other programs such as reproductive and child health and AIDS control program. Respondents felt the burden of other diseases to be much higher and overlooked the addressing risk factors like tobacco control as a real priority in the present context.

*We (have) major health programs comparing (compared) to these (tobacco control) programs, the tobacco control program is very small.*

*I think they (government) are not taking it (tobacco control) as a big issue.*

Tobacco, being a low priority issue, is a concern not only in India but also in many other developing and even developed countries [5, 23–25]. A study conducted in Australia regarding the translation of the tobacco control policy into practice found that tobacco was considered as a low priority issue and therefore the impact of policy was insignificant [26].

*(4.2) Lack of Resources.* Program managers reported lack of resources as the biggest hurdle for effective implementation of the program. This was reflected from the respondent's perspective on inadequacy of both financial support and manpower. Similar barriers of finances and costs have also been highlighted in other Indian studies and various other studies conducted in different parts of the world [27–30].

Studies from developing [30] and developed countries [25, 27] suggest similar barriers such as shortage of human resource and the shortage of information, education, and communication materials hampering effective implementation of tobacco control policy and program.

*We (program managers) need funds, manpower, material.*

Few respondents attributed lack of motivation and skills of the staff as major concerns for effective implementation of tobacco control policy and program. One of them stated the following:

*No new human resources needed, present human resources are enough, we [stakeholders'] have to motivate them [Human resource and staff] and develop their skills and utilize them.*

*(4.3) Lack of Monitoring and Interdepartmental Coordination.* Another significant barrier as reported by a few respondents was lack of interdepartmental coordination and motivation within the government. Few respondents also revealed lack of regular monitoring to be a major factor responsible for limiting implementation of tobacco control policy. For instance,

*No monitoring and supervision is done, strict implementation is not there.*

Two respondents also reported existing corruption within the system and political interference during the implementation of the policy as a barrier for effective implementation. For example,

*There are many problems, like corruption.*

*Once the policy [Smoke-free policy] is made then there should not be politician interference.*

Several cases urged for a need of adequate and comprehensive training in order to raise self-confidence and motivation amongst health care providers. Similar observations were made in other Indian studies which underscore the need of building capacity of health care providers and program implementers in tobacco control [5, 30].

The response of the cases on various aspects of implementation of tobacco control program was captured. It is clear from the number of positive responses received that there has been undoubtedly a lot of success achieved in implementation of tobacco control policy and program. On the other hand, various existing gaps and issues continue to hinder its successful implementation. This can be interpreted as negative responses. There are a very small percentage of cases that were neutral to certain issues of implementation of program. This clearly indicates that there exists a huge scope of improvement in implementation of tobacco control policy and program by overcoming the barriers. This is evident from a varied set of responses on each theme (Box 1).

## 4. Limitations

The study is limited by the fact that the views of program managers interviewed may not be generalizable to the health system in different states in India. The study was conducted over a short time frame. The results of the study should be viewed as a “snapshot” pertaining to just one time point when the views of stakeholders on tobacco policy and program implementation were captured. However, despite the inherent limitations, stakeholder analysis is a vital tool in informing the design of health systems research.

## 5. Conclusions and Policy Implications

The findings of this study obtained from “case analysis” approach captured perceptions of various program managers on several aspects of implementation of tobacco control policy and program. Respondents perceive lack of intersectoral coordination and resources, low prioritization of tobacco control in health and development programs, and lack of monitoring and evaluation of implementation of smoke-free laws as limiting factors affecting successful implementation of tobacco control policy and program. Some of the potential solution levers as suggested by many program managers include employing dedicated staff, regular training, and both financial and nonfinancial incentives to raise their motivation.

Based on the findings above, we highly recommend several actions to enable us to move towards significant reductions in the growing tobacco burden. First, we encourage central and state public health organizations to establish stringent and pragmatic goals which will help reduce tobacco use especially at the district level. At the same time, realistic planning needs to take into account existing capacity of the health system. An assessment of the health system implications and related resource costs will help in planning for proper implementation of tobacco control policy and program. This will also facilitate integration of the tobacco control program into broader national health plans.

## Figures and Tables

**Box 1:**
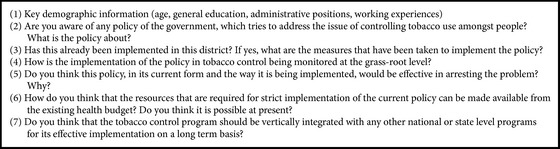
Discussion guide for capturing senior health program manager's perception.

**Table 1:** Key emergent themes and responses (stages 1 and 2).

Key emergent themes	Response of cases (*n* = 72)		No/neutral response
	Positive	Negative	
(1) Awareness on socioeconomic/financial/health impact (Positive = 42)	All the respondents responded positively to the awareness of ill effects of tobacco use *Yes it is financially burden for the people* *It is economic burden and also health problem* *TB, Cancer, Liver disease, digestion related problems, mouth Cancer, brain-tumor, Lung Cancer and those who have BP, Sugar will (suffer from heart) attack…*		

(2) Perception on effective implementation of the smoke-free laws Positive = 25 Negative = 10 Neutral = 7	Majority of the respondents were familiar with the laws and regulations towards tobacco use *Regularities of the 2003 policy and no smoking in public places* *If they smoke in public places if it is for first time acts 100 Rs fine and second time 200 RS*	Very few respondents were unaware of the policy per se but were familiar with the norms of the policy *Don't have that much idea about that policy* *Don't know about policies*	Few cases viewed lack of public support from the masses as an important barrier in implementation of the law *Can't say much about it because policy are just policies because people just read it and forget they* *No they (people) don't feel at all that tobacco control is their responsibility*

(3) Integration and its effectiveness Positive = 37 Negative = 5	Most respondents responded positively to the idea of integration of tobacco control program within other health programs *By integrating with that (NGO) we can get support like in technical and financial support. It will be better* *Integrating with NRHM gives more profit*	Few respondents raised concern about the idea of integration *If we give 10 programs likes that (together) it (tobacco control issue) gets diluted* *It should be separate program not integration* *It should be individual program*	

(4) Organizational Barriers			
(4.1) Tobacco: lack of prioritization Positive = 42	All the participants agreed that tobacco control lacks prioritization *The tobacco control program is very small* *I think they (government) are not taking it (tobacco control) as big issue*		
(4.2) Lack of resources Positive = 41 Negative = 40	Majority of the respondents recognized lack of resources to be a barrier *Yes there are no human resources* *We need funds, man power, material* *Lack of supervision and motivation, lack of self-motivation also* We don't have awareness camps here *No monitoring and supervision is done, strict implementation is not there* *There are many problems, like corruption*	One respondent denied the lack of resources as barriers to effective implementation of tobacco control program *No new human resources needed, present human resources are enough*	
(4.3) Lack of monitoring and interdepartmental coordination Positive = 42	All the respondents recognized lack of monitoring and coordination as a barrier *No monitoring and supervision is done, strict implementation is not there* *There are many problems, like corruption* *Once policy (smoke-free policy) is made then there should not be politician interference*		
